# Does the Public Prefer Health Gain for Cancer Patients? A Systematic Review of Public Views on Cancer and its Characteristics

**DOI:** 10.1007/s40273-017-0511-7

**Published:** 2017-04-29

**Authors:** Liz Morrell, Sarah Wordsworth, Sian Rees, Richard Barker

**Affiliations:** 1 Centre for the Advancement of Sustainable Medical Innovation (CASMI), University of Oxford, Room 4403, Level 4, John Radcliffe Hospital, Headley Way, Headington, Oxford, OX3 9DU UK; 2 Nuffield Department of Population Health, Health Economics Research Centre, University of Oxford, Oxford, UK; 3 Oxford NIHR Biomedical Research Centre, University of Oxford, Oxford, UK; 4 Nuffield Department of Primary Care Health Sciences, Health Experiences Institute, University of Oxford, Oxford, UK

## Abstract

**Background:**

Policies such as the Cancer Drugs Fund in England assumed a societal preference to fund cancer care relative to other conditions, even if that resulted in lower health gain for the population overall.

**Objective:**

The aim of this study was to investigate the evidence for such a preference among the UK public.

**Methods:**

The MEDLINE, PubMed and Econlit electronic databases were searched for studies relating to preferences for prioritising cancer treatment, as well as studies relating to preferences for the characteristics of cancer (severity of disease, end-of-life). The searches were run in November 2015 and updated in March 2017. Empirical preference studies, studies of public views, and studies in English were included.

**Results:**

We identified 24 studies relating to cancer preferences. Two directly addressed health trade-offs in the UK—one showed a preference for health gain in cancer, while the other found no such preference but provided results consistent with population health maximisation. Other studies mostly showed support for cancer but did not require a direct health trade-off. Severity and end-of-life searches identified 12 and 6 papers, respectively, which were additional to existing reviews. There is consistent evidence that people give priority to severe illness, while results for end-of-life are mixed.

**Conclusion:**

We did not find consistent support for a preference for health gains to cancer patients in the context of health maximisation. The evidence base is small and the results are highly sensitive to study design. There remains a contradiction between these findings and the popular view of cancer, and further work is required to determine the features of cancer which contribute to that view.

**Electronic supplementary material:**

The online version of this article (doi:10.1007/s40273-017-0511-7) contains supplementary material, which is available to authorized users.

## Key Points for Decision Makers

**Table:** 

Policies such as the Cancer Drugs Fund in England assume there is a societal preference to fund cancer care relative to other conditions.
This review finds that although the public consistently sees cancer as ‘special’, in the small number of studies that present respondents directly with a health trade-off, the results do not consistently support a preference for health gains in cancer.
There may be specific attributes of health gain within the ‘cancer’ label that are highly valued by the public and should be considered in decision making, in a way that is not disease-specific.

## Introduction

Cancer has been described as “the emperor of all maladies” [1], and despite significant improvements in survival rates for many cancers [2], it is still a ‘dreaded’ disease [3]. There are instances of health policies assuming that there is a preference for society to fund cancer care, relative to other diseases and conditions. For example, in the documents establishing the original Cancer Drugs Fund (CDF) in England, there is an assumption that the public value health gains to cancer patients up to twice as much as other conditions [4]. The CDF is unique in providing ring-fenced funds for a named disease, although there are examples of funds to cover specific types of conditions, such as the New Medicines Fund in Scotland, which supports access to drugs for end-of-life or rare conditions [5, 6]. The end-of-life criteria used by the National Institute for Health and Care Excellence (NICE) also reflect an assumed preference for health gain to patients with limited life expectancy [7], a feature of many cancers.

Health economic analysis typically assumes that the primary role of publicly funded healthcare is to maximise population health [8]. This is operationalised in cost-effectiveness analysis by assuming that ‘a quality-adjusted life-year (QALY) is a QALY’, i.e. a given level of health gain is equivalent regardless of the person it accrues to; it does not generally consider aspects outside the specific definition of health used for assessment, such as characteristics of the patient, the intervention or the condition itself [9, 10]. However, there are some particular circumstances that can be given generous weighting in NICE’s Technology Appraisal Committee deliberations, including severity of disease, end of life, and illnesses in children, and these aim to reflect societal preferences for allocation of healthcare resources [11].

Giving a preference weighting to cancer, or indeed any specific feature of ill health, requires understanding the trade-off involved: does society value health gains to cancer patients more highly than gains to other patients? More specifically, are we prepared to divert resources to cancer treatment even if it results in lower health gains for the population as a whole? Prioritising one disease type in this way within a fixed budget means that health is foregone by other patients within the population; hence, it has been argued that a strong case must be made to depart from the principle of health maximisation, and that this should reflect society’s views [12]. Therefore, our study aimed to explore the empirical evidence for a preference among the UK general public for health gain to cancer patients. Preliminary work indicated that limited empirical data exist specifically for cancer in the UK. To make our review more informative, we therefore chose to also look at similar data from other countries, and to consider proxies for cancer, in order to place the UK findings in context and enrich our interpretation; our focus, however, remains on the UK and cancer.

## Methods

A literature review was undertaken to identify empirical studies examining societal preferences for health gain to cancer patients. A search of the MEDLINE and PubMed electronic databases was conducted during November 2015, and updated in March 2017, using search terms covering both social preferences and cancer. In addition, a search of MEDLINE was conducted for the specific types of studies that would be used to address such preferences, such as discrete-choice experiments (DCEs). EconLit searches were added in March 2017. The search strategies are reported in Online Resource 1, section A. The terms referring to societal views were restricted to the title field to select papers with a direct focus on this topic.

Papers were screened by review of the abstracts, and eligible papers identified by full-text review (by LM). Studies were included if they were empirical studies of preferences for treating cancer patients relative to other conditions, studies of public views (i.e. excluding studies of clinicians and decision makers), and written in English; unpublished papers were not included.Study authors for the UK papers directly addressing health trade-off were contacted for points of clarification. The main data extracted from the studies (by LM, reviewed by SW) were the measure of preference for cancer treatments, its value, and whether a preference was demonstrated, along with key study features. Potential sources of bias for key papers were considered and are described in the Discussion section.

We also explored the literature on preferences for notable features of cancer, specifically severity (i.e. an illness that places patients in a poor health state) and end of life (where a patient’s life expectancy is short as a result of their illness). Both of these are used as prioritising features within health technology assessment, and often as proxies for cancer. While conclusions from studies in severity and end-of-life preferences do not necessarily apply to all cancers, many types of cancer will fall into at least one of these categories, therefore such studies could help support our understanding of cancer preferences. This area has been reviewed by Dolan et al. [13], Shah [14] and most recently by Gu et al. with searches run in August 2014 [15]. These were supplemented with MEDLINE and PubMed searches in February 2016 for additional publications since that work, with an update and Econlit searches conducted in March 2017 (details in Online Resource 1). Inclusion criteria were as above, replacing cancer with severity or end of life.

This report is consistent with the Preferred Reporting Items for Systematic Reviews and Meta-Analyses (PRISMA) reporting guidelines.

## Results

### Identified Studies

**Fig. 1:**
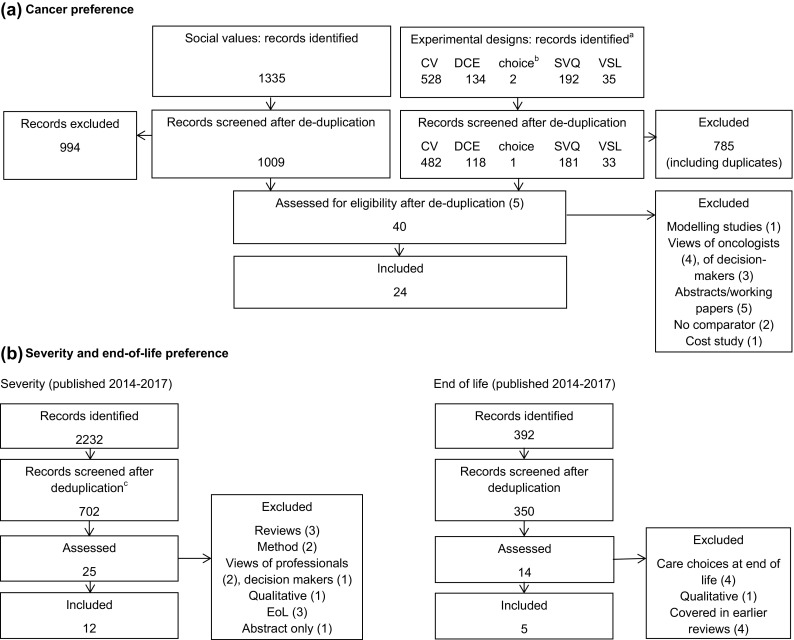
Literature search results. *CV* contingent valuation, *DCE* discrete-choice experiment, *SVQ* social value of a quality-adjusted life-year QALY, *VSL* value of a statistical life. ^a^Experimental design searches were run separately and then combined after deduplication and screening. ^b^Choice-based experiments were included as a search term to pick up choice-based designs other than the typical methods such as DCE. ^c^All MEDLINE and Econlit records plus the first 400 of 1868 from PubMed sorted by relevance

The literature search identified 24 empirical studies of public preferences for treating cancer (Fig. 1). Among the records recovered by the experimental method searches, the majority use contingent valuation (i.e. willingness to pay [WTP]); the initial high number of ‘social value of a QALY’ records were largely cost-effectiveness analyses rather than attempts to value health gain itself.

An overview of the papers is presented in Table 1 (further details are provided in Online Resource 1, sections B–D, with information on the 40 articles assessed in full). All except two papers are stated preference experiments where respondents are asked to respond to a hypothetical situation; Rojas [16] used experienced utility by asking respondents about their life satisfaction, while Gayer et al. [17] used a revealed preference approach to estimate the value of cancer risk reduction as reflected in house prices. Just over half of the studies (14/24) take a personal perspective; ten take a socially inclusive perspective, i.e. asking for preferences for a population of which they could be a member.

**Table 1:** Studies of public preference for treating cancer

Author	Year	Sample size	Method	Perspective^a^	Preference for cancer?^b^	Preference for cancer under explicit health gain trade-off?
Adamowicz et al. [28]	2011	1219	DCE and CV: WTP, VSL and VSI for water treatment	Socially inclusive, ex ante	√ 1*, 2*	– (Trade-off is with other personal consumption)
Alberini and Scasny [29]	2011	1906 (Italy), 1506 (Czech Republic)	DCE: VSL from WTP for air pollution reduction	Personal, ex ante	√ 1*	– (Trade-off is with other personal consumption)
Allen et al. [26]	2014	769	DCE: attributes of rural healthcare facilities	Personal, ex ante	√ 4*	– (Availability of service, not health gain)
Chestnut et al. [30]	2012	885 (US) 641 (Canada)	DCE, CV: WTP for preventive healthcare programmes	Personal, ex ante	X 2^, 4^	– (Trade-off is with other personal consumption)
Chim et al. [21]	2017	3080	Choice experiment	Socially inclusive, ex ante	X 3*	X 3*
Erdem and Thompson [18]	2014	250	DCE: WTP by marginal rate of substitution	Socially inclusive, ex ante	√ 2^#^, 4^#^	√ 2^#^, 4^#^
Gayer et al. [17]	2002	–	VSL by revealed preference	Personal, ex ante	X 1 ^#^	– (Trade-off is with other personal consumption)
Guignet and Alberini [31]	2015	2369 (Italy) 2426 (UK)	CV: VSL from WTP for house to reduce risk of pollution-caused death	Personal, ex ante	√ 1* (It) X 1^ (UK)	– (Trade-off is with other personal consumption)
Gyldmark and Morrison [22]	2001	948	CV: WTP to retain coverage	Personal, ex ante	√ 2^#^	– (Trade-off is with other personal consumption)
Hammit and Haninger [32]	2010	2018	DCE: WTP for food grown with safe pesticide	Personal, ex ante	X 2^	– (Trade-off is with other personal consumption)
Hammitt and Liu [33]	2004	1248	CV: WTP for intervention to reduce environmental pollutant	Socially inclusive, ex ante	X 1^, 2^#^	– (Trade-off is with other personal consumption)
Linley and Hughes [20]	2013	4118	Choice experiment	Socially inclusive, ex ante	X 3*	X 3*
McDonald et al. [34]	2016	157	Risk-risk trade-off: relative VSL	Personal, ex ante	√ 4*	– (Choice of least preferred type of death, not health gain)
Muhlbacher et al. [27]	2016	3900	DCE: attributes of health delivery system, including out-of-pocket costs	Personal, hypothetical ex post	√ 4^#^	– (Trade-off is with other personal consumption)
Neumann et al. [25]	2012	1463	CV: WTP for diagnostic	Personal, ex ante	√ 2*, 4*	– (Trade-off is with other personal consumption)
O’Shea et al. [23]	2008	435	CV: WTP to expand provision	Socially inclusive, ex ante	√ 2^#^, 5^#^	– (Trade-off is with other personal consumption)
Rojas [16]	2009	1000	Subjective well-being and marginal rate of substitution	Personal experienced utility, ex post	√ 2^#^	–
Romley et al. [24]	2012	270	CV: WTP for insurance coverage	Personal, ex ante	√ 2^#^	– (‘High-cost drugs’, not cancer-specific)
Savage [35]	1993	1027	CV: WTP for research to reduce mortality risk	Personal, ex ante	√ 2^#^	– (Trade-off is with other personal consumption)
Schomerus et al. [39]	2006	1012	Preferences for budget cuts	Socially inclusive, ex ante	√ 5^#^	–
Stegeman et al. [40]	2014	2946	Acceptance of behaviour-based differential access	Socially inclusive, ex ante	√ 3^#^	–
Tekesin and Ara [36]	2014	1248	DCE: VSL from marginal rate of substitution	Socially inclusive, ex ante	√ 1*, 2*	– (Trade-off is with other personal consumption)
Tsuge et al.[37]	2005	400	DCE: WTP by marginal rate of substitution	Personal, ex ante	√ 4*	– (Trade-off is with other personal consumption)
Viscusi et al. [38]	2014	3430	CV: VSL estimated from prevention cost	Personal/socially inclusive, ex ante	√ 1^#^	– (Trade-off is with other personal consumption)

*DCE* discrete- choice experiment, *WTP* willingness to pay, *CV* contingent valuation, *VSL* value of a statistical life, *VSI* value of a statistical illness, √ indicates evidence of a preference, *X* indicates no evidence of preference, – indicates not tested, * indicates statistically significant, ^ indicates not statistically significant, ^#^ indicates statistical testing of cancer differential not reported

^a^ Perspectives classified according to Dolan et al. [74]. Studies were designated as socially inclusive rather than social unless explicitly stated that the respondent was not personally at risk, and as ex ante unless explicitly stated that they need the intervention

^b^ The parameter used to indicate preference is represented as: 1, VSL; 2, WTP; 3, proportion; 4, regression coefficient; 5, ranking

Initial analysis identified three papers that directly addressed trade-offs between health gains to cancer patients and other use of resources; two of these studies were in the UK, and are described below. Studies outside the UK, or where respondents did not face this trade-off or considered aspects other than treatment, are discussed briefly.

### Trade-Off Studies

Erdem and Thompson [18] used a DCE to measure public preferences for attributes of healthcare innovations. In a DCE, respondents are asked to choose between two or more hypothetical scenarios, described in terms of defined attributes, which are set at various levels; choices are analysed to determine the extent to which each attribute contributes to the likelihood of a scenario being preferred. In that study, respondents were asked to choose which of two innovations their local National Health Service (NHS) should fund, described in terms of six attributes, one of which was the target population (levels: disability, cancer, mental health, obesity, asthma and drug addiction).

The study showed a significant preference for interventions for cancer patients (being a cancer intervention increased its likelihood of being chosen), and this was consistent across three clusters identified in a latent class analysis (a statistical technique to identify groups of respondents showing similar response patterns). By including cost as one of the parameters, the authors estimated WTP for the preferred characteristic, and, in the case of cancer, this was approximately £40 per month in extra taxation. This was consistently the highest among the conditions tested, across all latent groups, although the absolute value of this estimate should be treated with caution because it can be sensitive to the framing of the question; for example, framing as a monthly cost may overestimate WTP compared to a one-off fee [18], and WTP can differ for introduction of a service compared to its retention [19].

The authors showed example scenarios where an intervention for cancer patients was not the most likely to be chosen, i.e. cancer was not preferred in the absolute but could be traded off against combinations of the other parameters, such as health gain and strength of evidence.

Linley and Hughes [20] tested a range of parameters that could be used for prioritisation, in a resource allocation exercise where participants acted as a social decision maker, and allocated treatment between two groups that differed on one parameter at a time. The parameters included cancer and others, such as severity, end of life and reliance on carers. Respondents were asked to divide resources between two groups of patients, under three scenarios: all else being equal; health gain trade-off (i.e. the patients with the test characteristic would improve in health a little, whereas the other group would improve considerably); and cost trade-off (test characteristic group costs twice as much to treat).

With all else being equal, the data showed no preference for the cancer patients, with the majority of respondents dividing resources equally. Some of the parameters showed a significant preference (e.g. severity, reliance on carers), therefore, in principle, the study design was able to pick up such preferences. Under health gain trade-off, there was a shift in preference towards the group with the greater health gain, i.e. consistent with maximising population health rather than a preference for health gain in cancer.

Outside the UK, Chim et al. [21] fielded a study in Australia very similar to that of Linley and Hughes [20], with comparable findings, notably no evidence for a preference for health gains in cancer.

### Other Studies

Of the remaining 21 papers, six explored respondents’ WTP for specified interventions, services or coverage. Gyldmark and Morrison (Denmark) [22] aimed to test the validity of a contingent valuation design for obtaining public values, and as a measure of demand. The study evaluated WTP in insurance premium to maintain coverage for four conditions (uterine cancer, mild hypertension, diabetes in the elderly, and a broken wrist); uterine cancer showed the highest WTP of the four conditions tested. O’Shea et al. (Ireland) [23] explored WTP via tax or voluntary contribution, for cancer pain relief, community services for the elderly, or mental health. Both cancer and the elderly projects showed higher WTP than mental health. Importantly, neither of these studies controlled for severity, therefore the valuations reflect respondents’ perception of the severity of the conditions and the likely health gain. Romley et al. (US) [24] estimated WTP in additional insurance premium for generous coverage of ‘specialty’ (i.e. high-cost) drugs. They identified a high premium, although this was not cancer-specific. Neumann et al. (US) [25] used a DCE to measure WTP for a diagnostic test, with no immediate treatment decision. Respondents were most likely to choose to test for cancer, and these tests showed the highest WTP; however, this valuation is for the test, not for any corresponding treatment. Allen et al. (US) [26] and Muhlbacher et al. (US) [27] studied the design of health delivery systems, with Muhlbacher et al. finding that out-of-pocket cost considerations were lower for lung cancer than diabetes or current health. The study by Allen et al. is more difficult to interpret, finding no significant impact on WTP for local services when adding cancer care, but a negative impact when adding diabetes care or physical therapy.

Twelve of the studies [17, 28–38] estimated the value of a statistical life (VSL) for cancers by measuring WTP for reductions in cancer risk in comparison with other conditions. Scenarios typically included reduction or avoidance of environmental pollution [17, 28, 29, 31–33, 38] and comparisons with road traffic accidents [29, 34–37]. These studies are part of a body of risk literature estimating the VSL premium for cancer, reviewed by Tekesin and Ara [36], which finds cancer premiums ranging from 0 to 200%. Our review identified similar mixed results, with seven of the studies showing an effect of cancer context [28, 29, 34–38], three showing no effect [17, 32, 33], and two showing mixed effects across health conditions or countries [30, 31].

While a VSL premium supports the idea of cancer ‘dread’, the values in these studies reflect the value of prevention rather than health gain to patients. The exception is the study by McDonald et al. (UK) [34], which uses risk-risk trade-off to separate the effects of the ‘cancer’ label, duration of morbidity, and delay between exposure and symptom onset (latency). Although they found some evidence that the cancer context increased VSL relative to road accidents, the effect was counterbalanced by morbidity and latency effects, such that a scenario of a generic cancer with a latency of 10 years or more, and a morbidity period of 12 months, showed no premium.

From a list of nine conditions, Schomerus et al. (Germany) [39] asked respondents to choose where cuts could be made; cancer was rarely chosen. While this finding is consistent with a view of cancer as ‘special’, the study did not ask respondents to make trade-offs between specified characteristics of the conditions.

Stegeman et al. (The Netherlands) [40] explored views on differential access to healthcare based on the disease, and the patient’s health behaviour. Although the findings show a readiness to protect cancer patients from access sanctions, the yes/no response used in the study did not involve making a trade-off.

Rojas (Costa Rica) [16] used subjective well-being scores in a regression analysis including five conditions and income to estimate the monetary value of the illnesses; cancer had the highest monetary value at US$2700 per month, with arthritis second at US$1000 per month. This valuation is interpreted as the financial compensation that would return the respondent to their state of well-being before disease onset, and is not equivalent to WTP for treatment.

### Cancer Related Characteristics: Severity

There is a body of evidence indicating that people will depart from health maximisation to prioritise severe illness, beginning with work by Nord (Norway) [41] and Ubel (US) [42] in the 1990s, followed by Green in the UK [43]. The majority of 19 studies reviewed by Gu et al. [15] found the public generally give priority to patients with severe disease, although the definitions of severity in the studies are different and the values found are highly sensitive to experimental design, with some suggestion of a threshold effect.

Our supplementary searches identified 12 further studies related to severity (Table 2, column 6). Two included data for the UK, and results are consistent with the findings above. Specifically, van Exel et al. [44] used a factor analysis method (Q-sort) in a European study and identified five prioritising factors, one of which related to severity combined with maximising health gains. Rowen et al. [45] found some support for severity (defined by the impact on health-related quality of life and life expectancy) as a possible weighting factor in health technology assessment, using a DCE in the UK. Of the 10 further studies (Canada [46–48], The Netherlands [49, 50], Australia [21, 51], Belgium [52], Japan [53] and Poland [54]), nine showed some level of support for prioritising by severity. Five of the 12 studies described heterogeneity of preferences, and five suggested preference for equal dispersion of gains.

**Table 2:** Recent preference studies relating to severity and end of life^a^

Author	Year	Sample size	Method	Perspective	Preference for severity?	Preference for end of life?
Chim et al. [21]	2017	3080	Choice	Personal, ex ante	Majority prefer to allocate money to severe rather than moderate; shifts towards moderate under effectiveness trade-off	Majority prefer to allocate equally, particularly under effectiveness trade-off
Kolasa and Lewandowski [54]	2015	97	PTO	Societal decision maker, ex ante	Heterogeneity: young prioritised on severity and capacity to benefit, but older people not prepared to trade off	–
Luyten et al. [52]	2015	750	DCE	What should be funded by government	Severity is significant in driving choice but less than patient characteristics and treatment effectiveness. Heterogeneity: preferences differ with respondent characteristics	
Richardson et al. [51]	2016	662	Relative social WTP	Societal decision maker, ex ante	Supports a severity effect, with a threshold; weighting varies with the condition’s description	–
Rowen et al. [45]	2016	3669	DCE	Which group the NHS should treat	Some effect of BOI but inconsistent	(Preference for end-of-life conditions^b^)
Shiroiwa et al. [53]	2016	1000	Choice	Societal decision maker, ex ante	Similar proportion preferred severe and equal sharing	–
		1000	DCE	Societal decision maker, ex ante	Preference for young, treatment over prevention, and severity	–
Skedgel et al. [46]	2015	656	DCE	Societal decision maker, ex ante	Aversion to poor final health state. Heterogeneity: two latent classes differ in preferences over initial health state	Aversion to short initial life expectancy
Skedgel et al. [47]	2015	604	DCE and CSPC	Societal decision maker, ex ante	Preference for lower initial utility	No preference over untreated life expectancy in DCE, preference for longer life expectancy in CSPC
Skedgel [48]	2016	1318	DCE and CSPC	Societal decision maker, ex ante	Preference for prioritising severe initial health status; aversion to prioritising good initial and poorer final health status	No preference over untreated life expectancy
van de Wetering et al. [49]	2015	1205	DCE	Societal decision maker	Higher proportional shortfall not preferred in total sample. Heterogeneity: one of three latent classes showed preference to treat patients with low remaining health	–
van de Wetering et al. [50]	2016	1001	DCE	Societal decision maker	Severity shows some preference but unstable to adding in other parameters	–
van Exel et al. [44]	2015	294	Q-sort	How healthcare decisions should be made	Five viewpoints, one of which is severity and health maximising	Five viewpoints, one of which is life preservation
Wouters et al. [57]	2017	46	Q-sort	Personal, ex ante	–	Three viewpoints, none of which support preference for health gains in terminally ill patients

*DCE* discrete-choice experiment, *CSPC* constant sum paired comparison, *BOI* burden of illness, *NHS* National Health Service, *WTP* willingness to pay, *PTO* Person Trade-Off

^a^ Studies on severity and end of life published since the reviews of Gu et al. [15] and Chamberlain [55]

^b^ The study by Rowen et al. [45] is included in the Chamberlain end-of-life review (although listed as the earlier draft of Brazier et al. [75]) and is therefore not discussed in this article

### Cancer-Related Characteristics: End of Life

There is a smaller evidence base exploring preferences for health gain for patients whose life expectancy is short as a result of disease, reviewed by Chamberlain [55] and Gu et al. [15], who, between them, covered 11 studies. They found mixed results, with six studies showing weak or no effect of end of life on preferences, four showing some effect, and one describing heterogeneity of views. Again, results are highly sensitive to study design. Six of those studies are from the UK, of which two showed support for preference for end-of-life treatments. Shah [56] also recently reviewed end-of-life preference and came to similar conclusions; the review provides details of selected studies and commentary on methodological challenges and future research.

Our end-of-life searches identified five additional studies, plus an additional study from the severity search, which also had end-of-life information (Table 2, column 7). Two studies used Q-sort; van Excel et al. [44] found a viewpoint relating to preservation of life in a study including the UK, but Wouters et al. [57] found no viewpoint supporting an end-of-life preference in The Netherlands. Three DCE papers from Canada [46–48], and a choice study from Australia [21], also found no preference for treating patients with a short life expectancy.

## Discussion

Respondents in these studies view cancer as ‘special’ and deserving prioritisation, and, in some contexts, showed increased WTP (tax or personally) for cancer care or prevention. However, when presented with the opportunity cost of that choice, the results are inconsistent. As such, the literature reviewed does not provide a strong body of evidence supporting preference for health gains in cancer *per se*, and gives no clear indication for a weighting factor. This finding is consistent with other authors’ reviews (e.g. Linley and Hughes [20], Chamberlain [55] and Shah [58]); this paper updates and systematically extends that work. Evaporation of a preference for treating cancer when faced with its opportunity cost was also demonstrated by Gold et al. in a qualitative study [59]. The impact assessment for the establishment of the CDF in 2010 also found little support for its assumption of a cancer preference, and the absence of a specific cancer preference in the Linley and Hughes paper is commonly cited in critiques of the CDF, including the Scottish and Welsh governments’ decisions not to implement similar funds [60, 61].

The evidence on severity suggests that the public show a preference for health gains to patients with severe disease; however, the support for a preference for health gains at the end of life is equivocal. The variability in these results illustrates the challenges of designing experiments to determine definitive weights for these parameters for use in Health Technology Assessments (HTAs), with the results being sensitive to framing effects (preferences shifting with different descriptions of the problem). Interpretation is further complicated by evidence of heterogeneity in attitudes within the population surveyed, raising questions of how to represent an overall societal view [56]. Examples of the use of severity in HTA include Sweden and The Netherlands (variable threshold), as well as France (as a dimension of clinical benefit assessment) [62]. End-of-life criteria are used in HTA by NICE and the Scottish Medicines Consortium, allowing more flexibility in the cost per QALY under specific criteria [5, 7].

As the two UK studies requiring a trade-off disagree on the role of cancer in public preferences, it is important to explore potential sources of bias. The studies differ on several design elements, which might contribute to the contradictory results, and we suggest there is an overall tendency for the study by Erdem and Thompson [18] to overestimate, and the study by Linley and Hughes [20] to underestimate, preference for cancer. First, complexity; these are difficult choices and it is possible that in the multiattribute DCE, respondents resort to simple decision heuristics [63], such as prioritising the cancer patient, to make the decision more manageable. Indeed the authors’ own further analysis indicated use of selection by aspects [64] and attribute non-attendance [65] in the responses, with cancer consistently attracting respondents’ attention. This suggests that respondents were not fully considering the trade-offs in the scenarios, which resulted in overestimation of coefficients and WTP for cancer. The Linley and Hughes study [20] describes the choice in a simple health gain scenario. It may be that this provides respondents with a stark picture of the implications of favouring a particular group, in which the opportunity cost implications are not acceptable to the majority of respondents.

Second, the choices permitted: the DCE requires an all-or-nothing choice between two options (and a ‘none’ option), whereas the single parameter choice experiment in effect offers an 11-point scale. A forced choice can overestimate the degree of preference, particularly if there is a uniform direction of preference among respondents, but only a minimal perceived difference between the options. The results reflect the number of respondents with a given preference, but not the extent of that preference. In contrast, the scale may underestimate preference because of central tendency bias (respondents under-using the extremes of a scale). A similar hypothesis was tested by Skedgel et al., who compared the results of a binary-choice DCE with constant-sum paired comparison (budget sharing) and found that allowing respondents to distribute a budget across two alternatives provided richer preference data [47].

Third, the DCE may be sensitive to the choice of comparator conditions, which include conditions that might be considered behaviour-related (such as obesity), and where cancer is the only one that is typically considered immediately life-threatening. Any bias is likely to be in favour of cancer in this context.

Finally, both studies are limited in some aspect of generalisability. Erdem and Thomson report a relatively small and localised study (250 respondents in West Yorkshire), which may not be broadly generalizable for the UK, although comparison with the 2011 census indicated the sample was similar to the West Yorkshire population. The Linley and Hughes study provides a simple choice scenario; however, in reality, healthcare prioritisation is complex and based on multiple criteria, as outlined by Erdem and Thompson, therefore the single-parameter approach may not generalise to complex decisions. The direction of potential bias here is non-obvious.

Despite the inconsistency of results in this specific resource allocation context, there is a strong response to cancer in many of the studies reviewed. This is consistent with dual-processing theories of cognition as outlined by Kahneman [66], who described one processing system as fast and intuitive (System 1), while the other is slower and more deliberate (System 2). Using this model, we can describe the immediate responses to questions on cancer as triggering System 1, responding based on fear and dread, with trade-off questions that require further consideration invoking System 2. Simple heuristics in complex scenarios may be dominated by System 1, and not checked by System 2 so long as the resultant choices are coherent and acceptable. A similar explanation was proposed by Robb et al. [3] for qualitative observations of respondents responding initially to cancer questions with dread, while also acknowledging significant improvements in outcomes. Shah et al. [67] also observed participants in qualitative research struggling to reconcile logical resource allocation decisions with their intuitive response. Although there have been recent critiques of Kahneman’s work [68], it provides a useful structure for exploring the implications of the study’s findings. For example, it may be that the application of accountability for reasonableness principles [69] by HTA agencies (e.g. NICE [70]) makes these processes inherently reliant on System 2 judgements, and this will contribute to decisions being seen as unacceptable by the public if they are responding based on System 1.There are also implications for preference research, including the need for researchers to be clear on which type of response they are aiming to measure, and careful consideration of language in the materials and questions presented to respondents, with prior qualitative work to identify ‘trigger’ words that could prompt use of a simple heuristic. Parallel qualitative work with survey respondents may also help to understand the basis for their choices, as described by Shah [56].

### Limitations and Further Research

At the review level, we suggest that the main risk of bias across studies is likely to be publication bias. It may be that negative results (i.e. showing no evidence of a cancer preference) have a lower probability of publication, hence this review would overestimate any preferences.

The review is limited by the small number of studies that address the question of the value of health gains in cancer directly. This probably stems from the focus in economic evaluation on generic measures of health to allow comparison across disease areas; standard textbooks recommend that studies valuing health states are not labelled with specific conditions, and the value tariffs in common use were generated using unlabelled health state descriptions [71]. The impact of named conditions on how the public values health states has been reviewed by Brazier et al. [71] and Rowen et al. [72], showing mixed results. The study by Rowen et al. finds a reduction in the values assigned to severe health states labelled as cancer compared with irritable bowel syndrome (IBS) or no label [72]. In a study not covered in these reviews, Mason et al. [73] used named conditions in a prioritising exercise and did not observe cancer behaving noticeably differently from the other conditions; however, they do comment that familiar conditions such as cancer tended to receive more extreme scores, with less familiar illnesses more likely to be ranked in the middle. Despite the inconsistencies between studies, it appears that the name of the condition can be important in how the public value a given state, which, by extension, could affect the value of any health gain. Focusing on disease-blind health state valuations may be obscuring such public preferences.

Our review is limited by its focus specifically on societal attitudes to cancer in the context of resource allocation and health maximisation; however, we cannot ignore the reaction to cancer seen in other types of studies. Our conclusions are rooted in a health maximisation paradigm, which does not explicitly consider broader aspects beyond health gain. An alternative view that maximised something other than health (for example, utility as in welfare economics) would use measures such as individual WTP, which can be considered as a holistic reflection of what is important to the individual. In our review, such studies showed a cancer preference, suggesting there are features associated with cancer that are not reflected in standard measures of health. This potentially leads to undervaluation of interventions in health technology assessment, not only in cancer but also in other diseases, if aspects that are valued by the public are not captured. We hypothesise that the apparent uniqueness of cancer may be in the particular combination of such features, including, for example, the impact on the family, or the value of hope. Further research is needed to explore characteristic features of cancer in comparison with other significant health conditions such as cardiovascular disease and dementia. Our future work aims to identify broader aspects of health outcome that contribute to the value that patients and the public attach to therapeutic interventions.

## Conclusions

This review did not find consistent support for a preference for health gains to cancer patients in the specific context of resource allocation. However, the evidence base is small due to the focus in health economics on condition-blind valuation, and the results are highly sensitive to study design. There remains a contradiction between the findings and the popular view of cancer. Further understanding of the features that contribute to that view may reveal additional aspects of outcome that are valued by the public and could be considered when technologies are evaluated for funding.

## Electronic supplementary material

Below is the link to the electronic supplementary material.

Supplementary material 1 (DOCX 53 kb)
