# Supplementary material for: Does the Public Prefer Health Gain for Cancer Patients? A Systematic Review of Public Views on Cancer and its Characteristics
Source: Pharmacoeconomics. 2017 Apr 29;35(8):793–804. doi: 10.1007/s40273-017-0511-7 (PMC5548817; doi:10.1007/s40273-017-0511-7)
Supplement: Supplementary file 1 — Supplementary material 1 (DOCX 53 kb) [file 40273_2017_511_MOESM1_ESM.docx]

**SUPPLEMENTARY MATERIAL**

**A. Search strategies**

1. Social attitudes and cancer

Medline:

1 ("social value*" or "social preference*" or "public preference*").ti.

2 ("societal view*" or "societal preference*" or "societal value*").ti.

3 1 or 2

4 cancer.ti,ab.

5 exp Neoplasms/

6 4 or 5

7 exp Resource Allocation/

8 3 or 7

9 6 and 8

Pubmed:

(((((social value[ti] OR social values[ti]) OR (social preference[ti] OR social preferences[ti]) OR (public preference[ti] OR public preferences[ti]) OR (societal value[ti] OR societal values[ti]) OR (societal views[ti]) OR (societal preference[ti] OR societal preferences[ti]) OR "resource allocation"))) AND (("neoplasms") OR cancer[tiab]))

EconLit:

ti,ab(social view* or social value* or social preference* or societal view* or societal value* or societal preference* or public view* or public value* or public preference*) AND ti,ab,su(cancer)

The Pubmed/Medline searches used a Title specification for social attitude terms, to constrain the searches to papers focusing on this topic, rather than using these words generally as part of the discussion. As a check that this conservative specification was not missing important papers, the Medline search was re-run using the Title and Abstract fields for these terms; 69 additional papers were returned, but none met inclusion criteria.

In the EconLit searches, few papers were initially found using Title only for these terms, so it was broadened to Title and Abstract for the review to ensure no relevant papers were missed.

2. Experimental methods and cancer

Medline:

1 cancer.ti,ab

2 exp Neoplasms/

3 “discrete choice”.ti,ab

4 1 or 2

5 3 and 4

6 (“contingent valuation” or “willingness to pay” or “WTP”).ti,ab

7 4 and 6

8 SVQ.ti,ab

12 “choice-based experiment”.ti,ab

13 4 and 12

14 (“value” and “QALY”).ti,ab

15 8 or 14

16 4 and 15

EconLit:

ti,ab,su(cancer) AND ti,ab("discrete choice")

ti,ab,su(cancer) AND ti,ab("contingent valuation" or "willingness to pay" or "WTP")

ti,ab,su(cancer) AND ti,ab(choice-based”)

ti,ab,su(cancer) AND ti,ab(“VSL” or "value of a statistical life")

ti,ab,su(cancer) AND ti,ab("SVQ" or("value" AND "QALY"))

3. Severity

Medline:

1 ("social value*" or "social preference*" or "public preference*").ti,ab.

2 ("societal view*" or "societal preference*" or "societal value*").ti,ab.

3 exp Resource Allocation/

4 severity.ti,ab.

5 ("severe" or "severely ill").ti,ab.

6 ("serious" or "seriously ill").ti,ab.

7 ("discrete choice" or "contingent valuation" or "willingness to pay" or "willingness-to-pay" or "wtp" or "EuroVaQ").ti,ab.

8 1 or 2 or 3

9 7 or 8

10 4 or 5 or 6

11 9 and 10

Pubmed:

(((social[Title/Abstract] OR societal[Title/Abstract] OR public[Title/Abstract]) AND (views[Title/Abstract] OR perspective[Title/Abstract] OR perspectives[Title/Abstract] OR preference[Title/Abstract] OR preferences[Title/Abstract] OR values[Title/Abstract])) AND (severe[Title/Abstract] OR severely ill[Title/Abstract] OR serious[Title/Abstract] OR seriously ill[Title/Abstract] OR severity[Title/Abstract] OR equity[Title/Abstract])) Filters: Publication date from 2014/01/01 to 2016/12/31

EconLit:

(ti,ab(social view* OR social value* OR social preference* OR societal view* OR societal value* OR societal preference* OR public view* OR public value* OR public preference*)

OR

ti,ab("discrete choice" OR "contingent valuation" OR "willingness to pay" OR "WTP" OR "euroVaQ" OR "Value of a statistical life" OR "SVQ" OR (value AND “QALY”)))

AND

ti,ab(severity OR severe OR "severely ill" OR serious OR "seriously ill")

DATES: 2014-2017

4. End of life

Medline:

1 (“end of life” or “end-of-life”).ti,ab

2 (“short*” or “reduced”).ti,ab

3 “life expectancy”.ti,ab

4 2 and 3

5 “terminal”.ti,ab

6 “life exten*”.ti,ab

7 1 or 4 or 5 or 6

8 ("discrete choice" or "contingent valuation" or "willingness to pay" or "willingness-to-pay" or "wtp" or "EuroVaQ").ti,ab.

9 ("social value*" or "social preference*" or "public preference*").ti,ab.

10 ("societal view*" or "societal preference*" or "societal value*").ti,ab.

11 exp Resource Allocation/

12 8 or 9 or 10 or 11

13 7 and 12

Pubmed:

(social[Title/Abstract] OR societal[Title/Abstract] OR public[Title/Abstract]) AND (view[Title/Abstract] OR views[Title/Abstract] OR perspective[Title/Abstract] OR perspectives[Title/Abstract] OR preference[Title/Abstract] OR preferences[Title/Abstract] OR values[Title/Abstract]) AND (end of life[Title/Abstract] OR end-of-life[Title/Abstract] OR terminal[Title/Abstract] OR short life expectancy[Title/Abstract] OR shortest life expectancy[Title/Abstract] OR life extending[Title/Abstract] OR life extension[Title/Abstract]) Filters: Publication date from 2014/01/01 to 2016/12/31

Econlit:

(ti,ab(social view* OR social value* OR social preference* OR societal view* OR societal value* OR societal preference* OR public view* OR public value* OR public preference*)

OR

ti,ab("discrete choice" OR "contingent valuation" OR "willingness to pay" OR "WTP" OR "euroVaQ" OR "Value of a statistical life" OR "SVQ" OR (value AND “QALY”)))

AND

ti,ab("end of life" OR "end-of-life" or "terminal" or "life exten*" or "short* life expectancy" or "reduced life expectancy")

DATES: 2014-2017

**B. ASSESSED ARTICLES: CANCER PREFERENCE/SOCIETAL PREFERENCE**

Green = included in original review White = original, screened but not included

Yellow = included in 2017 update Yellow highlight = 2017, screened but not included

| Author | Year | Country | Sample | Approach | Result |
| --- | --- | --- | --- | --- | --- |
| Erdem & Thompson | 2014 | UK | General public, Yorkshire, postal | DCE including target population as one of the attributes (cancer as a level) | Preference for cancer in the sample as a whole and across 3 latent classes; WTP ~£40/month in extra tax. But not a golden bullet |
| Linley & Hughes | 2013 | UK | General public, internet, 4118 adults | Choice experiment: all things being equal, effect trade-off, cost trade-off | Significant majority (64.1%) shared equally for cancer/non-cancer. Some parameters eg severity, reliance on carers, showed preference. Shifts to non-cancer with effect trade-off, and to cancer with cost trade-off, both consistent with other parameters tested |
| O’Shea et al | 2008 | Ireland | Citizens | WTP tax or voluntary contribution for community based care in mental health, cancer, and elderly | Cancer and elderly WTP higher than mental health; cancer ranked highest importance |
| Rojas | 2009 | Costa Rica | Nationally representative survey | Monetary Value of Illness from Subjective Well-Being and income, for cancer, cardiovascular, thyroid, arthritis and infectious disease | Cancer had the greatest negative impact on most satisfaction domains, and overall Life Satisfaction. Also greatest monetary value in % and absolute terms, U$2700 vs closest arthritis, U$1000. |
| Schomerus et al | 2006 | Germany | National adult telephone survey | Preferences for cuts to be made in a list of 9 conditions; characteristics of the conditions | Cancer least chosen for cuts, mostly psych. Perceived as severe and life threatening. Life-threatening mostly not sig, but severity mostly is (but cancer not included in this analysis |
| Stegeman et al | 2014 | Netherlands | People who had entered a CRC screening programme, and tested negative | Differentiation in access for three conditions, based on a person’s choices – yes/no to differentiation by 3 mechanisms | Colorectal cancer less open to differentiation than COPD. Lung cancer matches COPD for insurance discount, and CRC for copayment and waiting list priority |
| Kwon et al | 2016 | South Korea | 300 members of the public | MCDA with 8 criteria including severity | Clinical benefits scored highest, then cost effectiveness and disease severity. Innovation the least preferred. *Doesn’t cover cancer but could include in severity* |
| Robertson et al | 2016 | Australia | 1318 randomly selected over 18 with a land line or mobile | Survey – quality of health care, concern about costs, contributors to cost. Compared to similar study in 2006 | New treatments for cancer more likely to be identified as contributor to cost, than in 2006. More likely to agree that patients should pay a greater proportion, less likely to agree that doctors should offer tx regardless of cost and chance of benefit. More agreed it was not the doctors job to be concerned about costs to society. *No comparison with other conditions, but maybe useful illustration that public understand that money is finite?* |
| Adamowicz et al | 2011 | Canada | 1219 representative online panel | Choice and contingent valuation: WTP to avoid illness or death from water contaminants via microbial illness or cancer | Microbial death has higher WTP than cancer, but once latency and discounting is included, cancer has the higher value.  Heterogeneity (latent classes) |
| Alberini & Scasny | 2010 | Italy | 1906 living in polluted cities | VSL: WTP for air pollution reduction.. | *Cancer as cause of death increases WTP by 1 million Euros. This is a working paper, written up in 2011 and 2013. Paper 2011 is a fuller source of the data* |
| Alberini & Scasny | 2011 | Italy, Czech | 1906(It), 1506 (Cz) public with children living in polluted cities | VSL: WTP in conjoint choice for air pollution reduction with cause of death as cancer, respiratory, or road accident | Cancer as cause of death increases WTP by 5 million (It) 1.8 million (Cz) Euros – with no ‘child premium’ |
| Alberini & Scasny | 2013 | Italy | 1906 living in polluted cities | Review, plus analysis of the Italian study from the other two papers | *Same data as 2011 paper* |
| Chim et al | 2017 | Australia | 3080 representative adults sample from a panel | Choice experiment: all things being equal, effect trade-off, cost trade-off | 57.6% shared funds equally between cancer and non-cancer. Parameters that were majority preference were severity, no other treatment on PBS, less well off, and lifestyle-unrelated. Shifts to non-cancer with effect trade-off, and to cancer with cost trade-off, both consistent with other parameters tested |
| Cropper et al | 2016 | US | 913 SurveyMonkey panel | Standard gamble: distribution of benefit for an environmental intervention | *Not about diseases, but equality of distribution* |
| Itaoka et al | 2007 | Japan | 1203 random sample, Tokyo and Osaka | Choice experiments: WTP for risk reductions from environmental pollution: lung cancer, bronchitis, pollen allergy | VSC: 313million Yen for lung cancer, 28.4 for bronchitis and 7.8 for allergy in a public good context, varying with discount rate. Private good: 812, 37, 15 *This is a working paper, not published or peer reviewed – excluded.* |

**C. ASSESSED ARTICLES: CANCER PREFERENCE/SPECIFIC EXPERIMENTAL METHODS**

Green = included in original review White = original, screened but not included

Yellow = included in 2017 update Yellow highlight = 2017, screened but not included

1. Contingent Valuation search

| Author | Year | Country | Sample | Approach | Result |
| --- | --- | --- | --- | --- | --- |
| Chestnut | 2012 | US, Canada | Internet panel samples | WTP by DCE, and payment card | Both designs: coefficients not significantly different for cancer vs heart |
| Greenberg et al | 2013 | Israel | Oncologists, family physicians | Stated preference, hypothetical patient (cancer or congestive heart failure), minimum improvement needed per increment, and WTP for a life extension. Derived $/QALY | Oncologists valued cancer life extension higher than for heart, and both were higher than GPs. QoL gains didn’t differentiate |
| Gyldmark & Morrison | 2001 | Denmark | National sample | WTP for insurance coverage for 4 conditions: mild hypertension, old persons’ diabetes, a broken wrist, and cancer of the uterus – if hypothetically, coverage was withdrawn ie Equivalent Variation | 105, 94, 78, 158 mean WTP in that order (DKK 2001). Mean max WTP followed the same order |
| Hammitt & Haninger | 2010 | US | Random sample of internet panel | WTP for food produced using safer pesticides | Cancer and organ did not have significantly different WTP |
| Hammitt & Liu | 2004 | Taiwan | Random dial phone survey | CV: WTP to reduce pollutant that can affect liver or respiratory. Condition either cancer, or failure/bronchitis | Cancer premium is not significant (p=0.12) |
| Neumann et al | 2012 | US | Residents | DCE of taking a test with no immediate treatment decision; disease, prevalence, accuracy and cost | Prostate cancer high, Alzheimer’s low |
| O’Shea et al | 2008 | Ireland | Citizens | WTP tax or voluntary contribution for community based care in mental health, cancer, and elderly | Cancer and elderly WTP higher than mental health; cancer ranked highest importance |
| Romley et al | 2012 | US | US adults | Stated preference, WTP for insurance coverage of specialty drugs ie “high-cost drugs that treat cancer and other serious conditions like MS” (didn’t separate the conditions) | $12.94 per month additional premium for “generous coverage” ie no copayment in the event that tx was needed. Cost of copayment estimated as $5/month. Higher income respondents less WTP |
| Savage | 1993 | US | Random dial phone survey, Illinois | WTP: share of $100 for research to reduce risk of stomach cancer, car accident, household fire, or aviation accident | Willingness to pay is increased by high dread and low knowledge – shown by stomach cancer |
| Tekesin & Ara | 2014 | Turkey | 4 cities in Turkey, general population | VSL relative to traffic accident – WTP in a DCE for mortality risk reduction from lung cancer, other cancer, respiratory disease, traffic accident | VSL ~0.5mill U$ 2012  Lung cancer premium of 213% against traffic accident; higher number for “other cancer” but not considered reliable  Order of coefficients: lung cancer, other cancer, resp disease, traffic |
| Thongprasert | 2015 | Thailand | 150 lung cancer patients, 150 members of the public never diagnosed with lung cancer | Score 2 vignettes; bidding game to estimate WTP to move from the worse to the better state. | WTP higher among the public than patients. Even among lowest income quartile, WTP was above the current Thai threshold of 1.2 GDP, and increased with income. *No comparison with other conditions* |
| Allen et al | 2014 | US | 769 random household sample in rural Kentucky | DCE 7 attributes of rural health care facilities: one is specialised care available – none, physical therapy, cancer care, and dialysis | Coefficient (and WTP) for cancer care is not significantly different from zero, where physical therapy (p<0.1) and dialysis (p<0.05) are negative. Strange result – perhaps public don’t expect to need them, irked at having to pay for something they don’t need |
| Cho | 2015 | Korea | 600 random sample from a representative panel | DCE to derive WTP in monthly premiums for extended coverage of liver cancer treatment | WTP of 2000 to 3333 KRW on monthly premium for a year’s full coverage of liver cancer treatment with simple models, and 4000-9000 KWR with added terms including gender, marital status, education.. *No comparison to other conditions* |
| Schwarzer | 2015 | multiple | 10 countries’ cost effectiveness thresholds | Comparison of thresholds with ability to pay | Moderate correlation with ability to pay. Identified alternative decision-making rules for oncology*. Decision makers not public* |

1. Discrete choice search

| Author | Year | Country | Sample | Approach | Result |
| --- | --- | --- | --- | --- | --- |
| Erdem & Thompson | 2014 | UK | General public, Yorkshire, postal | DCE including target population as one of the attributes (cancer as a level) | Preference for cancer in the sample as a whole and across 3 latent classes; WTP ~£40/month in extra tax. But not a golden bullet |
| Mulbacher et al | 2016 | US | 3900 individuals over 18 with access to the Duke University Healthview platform | DCE split into 4, with 21 attributes of health delivery systems, including out-of-pocket payments. Respondents randomly assigned to consider their current health, or hypothetical recently diagnosed with diabetes, or lung cancer | Mean relative importance estimate for out-of-pocket costs was lowest in the lung cancer scenario – other attributes were similar across the scenarios. (Out-of-pocket costs was most important parameter overall across all DCEs and scenarios) |

1. Choice-based experiment search

| Author | Year | Country | Sample | Approach | Result |
| --- | --- | --- | --- | --- | --- |
| Linley & Hughes | 2013 | UK | General public, internet, 4118 adults | Choice experiment: all things being equal, effect trade-off, cost trade-off | Significant majority shared equally for cancer/non-cancer. Some parameters eg severity, reliance on carers, showed preference. Shifts to non-cancer with effect trade-off, and to cancer with cost trade-off, both consistent with other parameters tested |

1. Value of a statistical life search

| Author | Year | Country | Sample | Approach | Result |
| --- | --- | --- | --- | --- | --- |
| Tekesin & Ara | 2014 | Turkey | 4 cities in Turkey, general population | VSL relative to traffic accident – WTP for mortality risk reduction from lung cancer, other cancer, respiratory disease, traffic accident | VSL ~0.5mill U$ 2012  Lung cancer premium of 213% against traffic accident; higher number for “other cancer” but not considered reliable  Order of coefficients: lung cancer, other cancer, resp disease, traffic |
| Viscusi et al | 2014 | US | Nationally representative | Annual increase in water cost to reduce risk of bladder cancer from arsenic contamination | Cancer premium 21% greater than for acute fatality for an immediate cancer risk. Notes that this premium is smaller than the 2x used by the HSE for cancer premium (as of 2001) |
| Guignet & Alberini | 2015 | UK, Italy | General public aged 40-60 living in cities. 2426 (UK) 2369 (Italy) | WTP for housing to reduce risk of pollution-caused death – cause of death randomly assigned as all causes, cancer, CV disease | Significant cancer premium in Italy of 75-85%, but not in the UK |
| Tsuge et al | 2005 | Japan | 400 randomly selected, Tokyo | VSL from DCE: purchase of a product to reduce mortality risk | No difference in VSL, although need to consider timing |
| Gayer et al | 2002 | US | House prices, newspaper words as proxy for risk perception | VSL cancer by revealed preference | Cancer VSL $4-3mill (national level), in line with other labour and product estimates |
| McDonald et al | 2016 | UK | 157 representative age 30-50, Newcastle | Risk-Risk trade-off: cancer and road accidents, latency and dread | No premium for generic cancer with morbidity period of 12 months and latency of 10 years or more. Latency counterbalances morbidity duration. |

1. Social value of a QALY search

| Author | Year | Country | Sample | Approach | Result |
| --- | --- | --- | --- | --- | --- |
| Bae & Mullins | 2014 | US | PubMed, QALY based ICERs 2003-13 | Calculated ICERs in oncology vs non-oncology (revealed preference) | Average ICER for cancer drugs >2x higher than non-cancer |
| Camps-Herrero et al | 2014 | Spain | 35 oncologists | “WTP” per QALY for new drugs | 30K-100KEuros per QALY 68.8% of sample |
| Drummond et al | 2014 | UK and France | NICE and HAS approvals 2003-2012 | Comparison of approval processes | Assessment of value largely agreed, but superior ASMR not associated with lower cost per QALY. Neither considered superior. |
| Greenberg et al | 2013 | Israel | Oncologists, family physicians | Stated preference, hypothetical patient (cancer or congestive heart failure), minimum improvement needed per increment, and WTP for a life extension. Derived $/QALY | Oncologists valued cancer life extension higher than for heart, and both were higher than GPs. QoL gains didn’t differentiate |
| Kozminski et al | 2011 | US | 1739 medical oncologists | Stated preference, hypothetical new chemo drug, compared life prolonging vs quality enhancing | $246K prolonging vs $119K quality, but with huge SD(>mean) |
| Nadler et al | 2006 | US | 139 academic medical oncologists in Boston | Gain in life expectancy to justify $70K a year hypothetical cancer drug, and estimates of cost and effectiveness of bevacizumab | $300K/QALY, only 25% think bevacizumab is value for money |
| Paramore et al | 2010 | US | (literature and government statistics) | Modelling: return on investment for biologic therapies, with health gain assumed to be valued at $100K/QALY. Asthma, diabetes and colorectal cancer. Expert panel estimates of impact | Range £207 (asthma) to $4 (CRC) per $ spent on the drugs |
| Thongprasert | 2015 | Thailand | 150 lung cancer patients, 150 members of the public never diagnosed with lung cancer | Score 2 vignettes of lung cancer; bidding game to estimate WTP to move from the worse to the better state. | WTP higher among the public than patients. Even among lowest income quartile, WTP was above the current Thai threshold of 1.2 GDP, and increased with income. *No comparison with other conditions* |

**D. DATA EXTRACTED FROM PAPERS: cancer, severity, end of life**

**1. Cancer**

| Author | Year | Country | Sample | Sample size | Method | Perspective | Comparators | Result | Limitations |
| --- | --- | --- | --- | --- | --- | --- | --- | --- | --- |
| Adamowicz et al | 2011 | Canada | Representative online panel | 1219 | DCE and contingent valuation: WTP for water treatment, derived VSL and VSI | Socially inclusive, ex ante | Microbial illness or death, cancer illness or death | Vs microbial, shows higher WTP (s) and VSI (s) for cancer illness, higher VSL for cancer death once latency is taken into account (s) although microbial death has higher WTP (ns).  Heterogeneity (latent classes) | Value of prevention rather than treatment |
| Alberini & Scasny | 2011 | Italy, Czech | Public with children, living in polluted cities | 1906(It), 1506 (Cz) | DCE: VSL from WTP for air pollution reduction | Personal, ex ante | Death from cancer respiratory, road traffic accident | Cancer as cause of death gives highest VSL, 5 million (s) (It) 1.8 million (s) (Cz) Euros – with no ‘child premium’ ie child VSL not different from adult VSL (ns) | Value of prevention rather than treatment |
| Allen et al | 2014 | US | Random household sample, rural Kentucky | 769 | DCE: attributes of rural health care facilities | Personal, ex ante | Specialised care available: none, physical therapy, cancer care, dialysis | Coefficient and WTP for cancer care not significantly different from zero, but physical and dialysis had significantly negative coefficients. Difficult to interpret in terms of preference | Difficult to interpret negative coefficients – expect something to be better than nothing! Does low willingness to pay suggest it’s sufficiently important to go to a main centre? |
| Chestnut et al | 2012 | US, Canada | Internet panel sample | 885 (US) 641 (can) | DCE, CV: derived VSL | Personal, ex ante | Heart, pneumonia | No sig difference in DCE coefficients but pneumonia coefficient ns. No difference in WTP by payment card (ns). Similar VSL between heart and cancer for DCE or payment card methods (overlapping CIs, ns) | Value of prevention rather than treatment |
| Chim et al | 2017 | Australia | General public, panel, online | 3080 | Choice experiment | Socially inclusive, ex ante | cancer, non-cancer | Significant majority (57.6, s) shared resources equally for cancer/non-cancer; shift to non-cancer with effect trade-off (s), and to cancer with cost trade-off | No named comparator.  Is the “all things being equal” believable in the context of cancer? |
| Erdem & Thompson | 2014 | UK | General public, Yorkshire; postal | 250 | DCE: WTP by marginal rate of substitution | Socially inclusive, ex ante | disability, cancer, mental health, obesity, asthma, drug addiction | Highest coefficient for cancer among the diseases, in the sample as a whole and across 3 latent classes; WTP ~£40/month in extra tax, highest among diseases. (differences nt for sig). But not a golden bullet | Effect of choice of comparators. Complex decision, risk of inattention? |
| Gayer et al | 2002 | US | House prices | 16928 house sales | VSL by revealed preference | Personal | Other VSL estimates | VSL for cancer $4.3million, in line with other estimates (sig nt) | Value of prevention rather than treatment |
| Guignet & Alberini | 2015 | UK Italy | General public age 40-60 living in cities | 2426 (UK), 2369 (It) | CV: VSL from WTP for house to reduce risk of pollution-caused death | Personal, ex ante | cause of death randomly assigned as all causes, cancer, CV disease | Significant VSL cancer premium in Italy of 75-85%, but ns in the UK | Value of prevention rather than treatment |
| Gyldmark & Morrison | 2001 | Denmark | National, adults; face-to-face | 948 | CV: WTP to retain coverage | Personal, ex ante | mild hypertension, old persons’ diabetes, broken wrist, cancer of the uterus | 105, 94, 78, 158 mean WTP in that order (DKK 2001) (sig nt). Mean max WTP followed the same order | Effect of choice of comparators. No trade-off with health gain as severity or gain not specified |
| Hammit & Haninger | 2010 | US | Random sample of internet panel | 2018 | DCE: WTP for food grown with safe pesticide | Personal, ex ante | Cancer, non cancer | No significant difference in WTP for cancer | Value of prevention rather than treatment |
| Hammitt & Liu | 2004 | Taiwan | Random dial phone survey | 1248 | CV: VSL from WTP for intervention to reduce environmental pollutant | Socially inclusive, ex ante | Liver and lung cancer, liver failure, bronchitis | Cancer premium is not significant: WTP is larger but ns, and minimal difference in derived VSL | Value of prevention rather than treatment |
| Linley & Hughes | 2013 | UK | National, adults; internet panel | 4118 | Choice experiment | Socially inclusive, ex ante | Cancer, non-cancer | Significant majority 64.1% shared resources equally for cancer/non-cancer; significant shifts, to non-cancer with effect trade-off, and to cancer with cost trade-off | No named comparator.  Is the “all things being equal” believable in the context of cancer? |
| McDonald et al | 2016 | UK | Public age 30-50, Newcastle | 157 | Risk-Risk tradeoff: relative VSL | Personal, ex ante | Cancer, road accident death | Cancer context significantly increases the relative VSL vs road accident, as shown by significant coefficient in some regression models, but is counterbalanced by latency and morbidity of cancer death; generic cancer with morbidity of 12 months and latency 10 years+ has no premium. | Not trading with a specified level of health gain |
| Mulbacher et al | 2016 | US | Adults with access to a Duke University health platform | 3900 | DCE: attributes of health delivery system, including out-of-pocket costs | Personal, hypothetical ex post | Current health, or hypothetical recently diagnosed with diabetes or lung cancer | Mean relative importance estimate for out-of-pocket costs was lowest (nt for significance) in the lung cancer scenario – other attributes were similar across the scenarios. (Out-of-pocket costs was most important parameter overall across all DCEs and scenarios) | Mix of hypothetical and real health states |
| Neumann et al | 2012 | US | National; internet panel | 1463 | CV: WTP for diagnostic that predicts future status | Personal, ex ante | prostate cancer, breast cancer, arthritis, Alzheimer’s | Cancer scenarios had significantly higher inclination to take the test (sig positive coefficient) and sig higher WTP, than arthritis | Value of test rather than treatment |
| O’Shea et al | 2008 | Ireland | National, adults; face-to-face | 435 | CV: WTP tax or voluntary contribution | Socially inclusive, ex ante | community based care in mental health, cancer, and elderly | Cancer and elderly WTP higher than mental health, across most characteristics (sig nt); cancer ranked highest importance by 62% | No matching of severity or health gain between scenarios |
| Rojas | 2009 | Costa Rica | Nationally representative survey | 1000 | Subjective Well Being: Monetary Value of Illness by marginal rate of substitution | Personal experienced utility, ex post | cancer, cardiovascular, thyroid, arthritis, infectious disease | Cancer had the greatest negative impact on most satisfaction domains, and overall Life Satisfaction. Also greatest monetary value in % and absolute terms, U$2700 per month vs closest arthritis, U$1000 (sig nt). | Monetary value is a measure of compensation needed to maintain constant Life Satisfaction with disease, rather than WTP to treat/cure the disease |
| Romley et al | 2012 | US | Nationally representative; internet panel | 270 | CV: WTP for insurance coverage of high-cost drugs | Personal, ex ante | no out-of-pocket payments, monthly out-of-pocket payments | $12.94 per month additional premium for full coverage (sig nt) | Addresses “high-cost drugs that treat cancer and other serious conditions like MS” rather than cancer specifically |
| Savage | 1993 | US | Random phone survey, Illinois | 1027 | CV: WTP for research to reduce mortality risk | Personal, ex ante | Stomach cancer, car accident, household fire, aviation accident | WTP higher for stomach cancer (sig nt) – low knowledge, and high dread (below car accidents) | Value of prevention rather than treatment. No trade-off with other health gain |
| Schomerus et al | 2006 | Germany | National, adults; telephone | 1012 | Preferences for cuts | Socially inclusive, ex ante | Cancer, myocardial infarction, diabetes, rheumatism, AIDS, Alzheimer’s, schizophrenia, depression, alcoholism | Cancer ranked lowest in choice for cuts. Perceived as severe and life threatening. | No trade-off with health gain |
| Stegeman et al | 2014 | Netherlands | Participants in a CRC screening programme; postal | 2946 | Acceptance of differentiation in access based on behaviour | Socially inclusive, ex ante | colorectal cancer, lung cancer, COPD | Lower proportion of agreement to differentiation for CRC (minority) than COPD (majority) (sig nt). Lung cancer matches COPD for insurance discount (reward), and CRC for copayment and waiting list priority (sanction) | No trade-off against health gain  Not a generalizable sample. |
| Tekesin & Ara | 2014 | Turkey | Adults in 3 cities; face-to-face | 1248 | DCE: VSL from marginal rate of substitution | Socially inclusive, ex ante | Risk reduction for traffic accident, lung cancer, other cancer, respiratory disease | Cancer coefficients and hence WTP sig greater than respiratory, and traffic accident sig less. Lung cancer premium in VSL of 213% against traffic accident. | Value of prevention rather than treatment |
| Tsuge et al | 2005 | Japan | Random sample, Tokyo | 400 | DCE: WTP by marginal rate of substitution | Personal, ex ante | Purchase of a product to reduce mortality risk – accident, cancer, heart disease, general causes | Significant positive coefficients for cancer risk in most models (highest preference for cancer | Value of prevention rather than treatment |
| Viscusi et al | 2014 | US | Nationally representative: internet panel | 3430 | CV: VSL estimated from increased water cost | Personal/ socially inclusive, ex ante | Risk of bladder cancer from arsenic contamination in water supply | Cancer premium: VSL 21% greater than for acute fatality for an immediate cancer risk (sig nt)  Notes that this premium is smaller than the 2x used by the HSE for cancer premium (as of 2001) | Value of prevention rather than treatment |

DCE: discrete choice experiment WTP: willingness to pay CV: contingent valuation VSL: value of a statistical life VSI: value of a statistical illness

s: statistically significant ns: not significant nt: statistical testing not reported

**2. Severity 2014-2017 – data extracted**

| Author | Year | Country | Sample | Sample size | Method | Perspective | Definition of severe | Result | summary |
| --- | --- | --- | --- | --- | --- | --- | --- | --- | --- |
| van Exel et al | 2015 | 10 Euro countries | General public: convenience samples, newspaper ads, agency | 294 | Q-sort | Personal, ex ante | Statements include worsening health, life threatening | 5 viewpoints, one is severity and health maximising | Y, heterog |
| Skedgel et al | 2015 | Canada | Public (internet panel) plus decision makers | 656 | DCE | Societal decision maker, ex ante | Initial health utility, scale 1/10 to 10/10 | Preference for younger, and wider distribution of gains. Aversion to short initial life expectancy and poor final health state. 2 latent classes ie heterogeneity. Pref for dispersion | no |
| Skedgel et al | 2015 | UK and Canada | Students (Sheffield, UK), students and staff (Halifax, CA), hospital staff (Halifax, CA) | 604 | DCE, CSPC | Societal decision maker, ex ante | Initial health utility, scale 1/10 to 10/10 | Preference for higher final utility (strongest in both designs), QALY gain, younger and lower initial utility. Preference for longer initial life expectancy in CSPC ie not EOL. No pref for dispersion. Suggests richer pref data in CSPC. | Y but low |
| Skedgel | 2016 | Canada | Public (internet panel) | 1318 | DCE and CSPC | Societal decision maker, ex ante | Initial health utility, scale 1/10 to 10/10 | Aversion to prioritising older patients, smaller groups, good initial and poorer final health status; preference for prioritising severe initial health status and larger groups. No preference over untreated life expectancy | Y |
| van de Wetering et al | 2015 | Netherlands | Adult, national, internet panel | 1205 | DCE | Societal decision maker, ex ante | Proportional shortfall, or remaining health without treatment | Higher proportional shortfall not preferred in total population. Latent class model (ie heterogeneity) identified one of 3 groups showing preference for treating patients with low remaining health | No, heterog |
| van de Wetering et al | 2016 | Netherlands | Adult, national, internet panel | 1001 | DCE | Societal decision maker, ex ante | QoL before treatment, scale 0-100 | Severity shows some preference unless very low, but unstable to adding in other parameters, and not monotonic in all scenarios. Aversion to small health gain.  Age 10 and 40 preferred to 70 and 90; against culpability, pro rare and dependants. | Yes, inconsistent |
| Luyten et al | 2015 | Belgium | National representative managed panel, internet | 750 | DCE | How the general populations thinks | Duration, effect on QoL, and lethality | Severity is significant in driving choice but lower coefficient than patient characteristics (age, lifestyle) and tx effectiveness. Heterogeneity and interactions observed – heterogeneity based on observed respondent characteristics, particularly age lifestyle health and education. | Yes but not the strongest driver |
| Rowen et al | 2016 | UK | General population, internet panel | 3669 | DCE | Which group they think the NHS should treat | Burden of illness (loss of HRQoL and life expectancy vs maximum) | Modest effect of BOI but inconsistent. Preference for end of life and QALY gain. Preference to disperse gains (diminishing marginal social value) | Yes, inconsistent |
| Shah et al | 2015 | UK | Representative internet panel | 3969 | DCE | Which patient they think should be treated | - | Greater influence of size of gains over life expectancy or initial QoL | no |
| McHugh et al | 2015 | UK | Purposive | 61 | Q-sort | Personal | - | 3 viewpoints: one is life preservation | Y, heterog |
| Chim et al | 2017 | Australia | Adult, national online panel | 3080 | Choice | Your preferred way for PBS to spend government money | severe health problems that affect patients’ well-being considerably | 52.7% preferred to allocate money to severe rather than moderate; shifts towards moderate in effect trade-off (45% now share equally, ~25% each severe/mod, but little effect of cost trade-off. | Yes but sensitive to health maximisation |
| Shiroiwa et al | 2016 | Japan | Web panel | 1000 | Choice | Societal decision maker, ex ante | 20/100 vs mild, 70/100 | 35.9% preferred severe, with 36.8% sharing equally. Age was the only attribute to show >50% preference (for younger patients) | Weak. Heterogeneity and preference to distribute |
|  |  |  | Random door-to-door | 1000 | DCE | Societal decision maker, ex ante | 20/100 vs mild, 70/100 | Age highest coefficient (younger) followed by treatment objective (vs prevention) and severity – p<0.0001 | y |
| Wouters et al | 2017 | Netherlands | General public, incl cancer patients | 46 (10 cancer experienced) | Q-sort | Personal, ex ante | - | - | - |
| Kolasa & Lewandowski | 2015 | Poland | Over 65, under 25 | 97 | PTO | Societal decision maker, ex ante | Initial health, 0-1 | Young prioritised on severity and capacity to benefit, and showed inequality aversion. Older people didn’t – not prepared to trade | Y among younger, |
| Richardson et al | 2016 epub | Australia | Representative on-line | 662 | Relative Social WTP | Societal decision maker, ex ante | Health states described by EQ5D5L dimensions and levels | Supports a severity effect, with a threshold, and the weighting varies with the type of condition (EQ5D dimensions, not disease) | Y |
| Kwon et al | 2017 | South Korea | Public | 300 | MCDA | ? | ? | Severity along with clinical effectiveness and cost effectiveness highest ranked | Y |

**3. Severity and End of Life 2014-2017**

| Author | Year | Country | Sample size | Method | Perspective | Result: severity | Result: end of life | Observations |
| --- | --- | --- | --- | --- | --- | --- | --- | --- |
| van Exel et al | 2015 | 10 Euro countries | 294 | Q-sort | Personal, ex ante | 5 viewpoints, one is severity and health maximising | 5 viewpoints, one is life preservation | Heterogeneity |
| Skedgel et al | 2013 (online, actual paper 2015) | UK and Canada | 604 | DCE, CSPC | Societal decision maker, ex ante | Weak preference for lower initial utility | (No significant preference for initial life expectancy in CSPC ie not EOL.) | No preference for dispersion |
| Skedgel et al | 2015 | Canada | 656 | DCE | Societal decision maker, ex ante | Aversion to poor final health state, no significant preference over initial health state | Aversion to short initial life expectancy, preference to prioritise longer | Preference wider distribution of gains.  2 latent classes ie heterogeneity; differ on preferences over initial health state. |
| Skedgel | 2016 | Canada | 1318 | DCE and CSPC | Societal decision maker, ex ante | Aversion to prioritising good initial and poorer final health status, preference for prioritising severe initial health status | No preference over untreated life expectancy | Paper compares public view with pCODR decision-makers, but they’re not relevant for this review |
| van de Wetering et al | 2015 | Netherlands | 1205 | DCE | Societal decision maker, ex ante | Higher proportional shortfall not preferred in total population. Latent class model identified one of 3 groups showing preference for low remaining health | - | 3 latent classes ie heterogeneity |
| van de Wetering et al | 2016 | Netherlands | 1001 | DCE | Societal decision maker, ex ante | Severity shows some preference but unstable to adding in other parameters, and not monotonic in all scenarios | - |  |
| Luyten et al | 2015 | Belgium | 750 | DCE | How the general populations thinks | Severity is significant in driving choice but lower coefficient than patient characteristics (age, lifestyle) and tx effectiveness. | - | Heterogeneity determined by interaction terms. Heterogeneity in preferences based on respondents’ characteristics: notably age, health, education, lifestyle |
| Rowen et al | 2016 | UK | 3669 | DCE | Which group they think the NHS should treat | Some effect of BOI but inconsistent | Preference for end of life | Preference to disperse gains (diminishing marginal social value) |
| Shah et al | 2015 | UK | 3969 | DCE | Which patient they think should be treated | - | Greater influence of size of gains over life expectancy or initial QoL | Heterogeneity |
| McHugh et al | 2015 | UK | 61 | Q-sort | Personal | - | 3 viewpoints: one is life preservation | Heterogeneity |
| Chim et al | 2017 | Australia | 3080 | Choice | Personal, ex ante | 52.7% preferred to allocate money to severe rather than moderate; shifts towards moderate in effect trade-off (45% now share equally, ~25% each severe/mod, but little effect of cost trade-off. | 49.7% prefer to allocate equally, shift toward equal allocation with effect trade-off although proportion favouring EOL remains the same. Cost trade-off shifts preference towards EOL | Preference to disperse gains relative to cost (shift to expensive to not disadvantage the high cost group) |
| Shiroiwa et al | 2016 | Japan | 1000 | Choice | Societal decision maker, ex ante | 35.9% preferred severe, with 36.8% sharing equally. | - | Preference to share equally |
|  |  |  | 1000 | DCE | Societal decision maker, ex ante | Age highest coefficient (younger) followed by treatment objective (vs prevention) and severity – p<0.0001 | - | Preference to share equally |
| Wouters et al | 2017 | Netherlands | 46 (10 cancer experience) | Q-sort | Personal, ex ante | - | 3 viewpoints, none of which support preference for health gains in terminally ill patents |  |
| Kolasa & Lewandowski | 2015 | Poland | 97 | PTO | Societal decision maker, ex ante | Young prioritised on severity and capacity to benefit, and showed inequality aversion. Older people didn’t – not prepared to trade | - | Young people showed inequality aversion – preferred equal health gain across 2 groups to double health gain for one |
| Richardson et al | 2016 epub | Australia | 662 | Relative Social WTP | Societal decision maker, ex ante | Supports a severity effect, with a threshold, and the weighting varies with the type of condition (EQ5D dimensions, not disease) | - |  |
| Kwon et al | 2017 | South Korea | 300 | MCDA | Societal and Personal, ex ante | Severity along with clinical effectiveness and cost effectiveness highest ranked | - | Full text not available |

Blue were in this search, are in Chamberlain review but not in Gu. Rowen is in Chamberlain for EoL (but as the Brazier et al EEPRU report) but not in Gu for severity or EOL. Papers already reviewed elsewhere are not included in our review.
